# The Role of Visual Processing Speed in Reading Speed Development

**DOI:** 10.1371/journal.pone.0058097

**Published:** 2013-04-04

**Authors:** Muriel Lobier, Matthieu Dubois, Sylviane Valdois

**Affiliations:** 1 Laboratoire de Psychologie et NeuroCognition, CNRS -UMR 5105, Université Pierre-Mendès-France, Grenoble, France; 2 Laboratoire Langage, Cognition et Développement, Université Libre de Bruxelles, Bruxelles, Belgium; 3 National Fund for Scientific Research, FRS-FNRS, Bruxelles, Belgium; University of British Columbia, Canada

## Abstract

A steady increase in reading speed is the hallmark of normal reading acquisition. However, little is known of the influence of visual attention capacity on children's reading speed. The number of distinct visual elements that can be simultaneously processed at a glance (dubbed the visual attention span), predicts single-word reading speed in both normal reading and dyslexic children. However, the exact processes that account for the relationship between the visual attention span and reading speed remain to be specified. We used the Theory of Visual Attention to estimate visual processing speed and visual short-term memory capacity from a multiple letter report task in eight and nine year old children. The visual attention span and text reading speed were also assessed. Results showed that visual processing speed and visual short term memory capacity predicted the visual attention span. Furthermore, visual processing speed predicted reading speed, but visual short term memory capacity did not. Finally, the visual attention span mediated the effect of visual processing speed on reading speed. These results suggest that visual attention capacity could constrain reading speed in elementary school children.

## Introduction

Typical children learn the basic mechanics of reading in one to two years. Reading speed, in contrast, increases continually from elementary school up to college [1]. While a large body of research points to the importance of linguistic factors in reading acquisition (see [2] for a review), the role of visual processing in word recognition fluency is still under debate. An important aspect of reading is the necessity to visually process several letters in a very short time frame (50 ms can be long enough for an expert reader to identify a word [3], [4]). It has therefore been suggested that simultaneous processing of letters in words is necessary for fluent reading [5], [6]. Indeed, the absence of length effects is a hallmark of expert reading [7]–[9]. Furthermore, even at very short presentation times, all letters in a word undergo at least partial processing [10]. Efficient visual word recognition could therefore depend on efficient parallel visual processing of multiple letters. This putative influence of the visual front-end of visual word recognition on reading performance can be investigated and modeled in two non-mutually exclusive ways. One approach is to specify how reading-specific, specialized processes can develop with reading acquisition and modulate reading performance. Another approach is to specify how general visual processing skills can play a role in visual word recognition and modulate reading performance.

According to the LCD model, parallel processing of individual characters in letter strings results from perceptual expertise for print [6]. The development of this perceptual expertise or tuning of the visual system is driven by perceptual learning mechanisms. Within the LCD framework, letter string processing is carried out by a hierarchical neural network of letter/letter combination detectors. In this network, higher-order, location independent, abstract letter detectors receive input from lower order, location dependent, case specific letter detectors. This highly structured network is thought to be located in the left ventral occipito-temporal cortex, in an area dubbed the visual word form area [11], [12]. Neural and behavioral evidence suggests that this perceptual expertise for print appears and develops with reading acquisition [9], [13]–[16]. Furthermore, it suggests that in addition to a general neural tuning for individual letters, expert reading is associated with a more specific perceptual specialization for horizontal letter strings [8]. The LCD model therefore posits that parallel visual processing of letters in letter strings results from the development of reading-specific perceptual skills.

Other studies have investigated the putative role of general visual processing skills on reading performance. Poor reading skills have been associated with poor performance on motion detection performance, most notably in dyslexic readers [17], [18]. Dorsal stream processing could thus play a role in reading acquisition. Recent data however suggests that the relationship between coherent motion detection and reading performance in typical children could be driven by the effect of age [19]. It has also been shown that visual spatial attention skills in pre-reading children predict future reading skills [20]. Nevertheless, the exact mechanisms through which visual spatial attention or dorsal stream functioning could impact reading efficiency have yet to be formally modeled.

According to the connectionist Multi Trace Memory model (MTM) of reading [5], visual attention capacity modulates reading performance. In this model, visual attention capacity is modeled by a visuo-attentional window. This visuo-attentional window determines the number of visual elements (sublexical units) that can be simultaneously encoded during reading. A larger visual attention capacity is associated with a larger number of visual elements (letters, graphemes, or syllables). Fast recognition of familiar words hinges on the window encompassing the entire letter string. In this case, all letters are encoded simultaneously. The entire letter sequence then activates previously encountered letter sequences. If a match is made, fast word recognition is achieved. However, if word identification fails or if the window cannot encompass the whole word, the system shifts to a slower mode of recognition. In this case, the number of elements the visuo-attentional window encompasses is progressively reduced to smaller and smaller units (syllables, graphemes, then letters) until a match can be made to long term memory traces. These shorter letter sequences are encoded and matched to long term memory traces serially until the entire word has been processed. Word recognition is then a serial and protracted process. The maximum size of the visuo-attentional window (i.e., visual attention capacity) limits visual word recognition speed. The MTM model therefore posits that non-reading specific visual skills could modulate reading performance.

Predictions of the MTM model have been tested in normal-reading [21], [22] and dyslexic children [23]–[26]. The visual attention (VA) span, defined by Bosse et al. [25] as the number of individual visual elements that can be processed simultaneously, operationalizes the visuo-attentional window. VA span is typically measured with letter report tasks: a string made up of five unrelated consonants is displayed for 200 ms and participants are asked to report either as many of the displayed letters as possible (whole report) or a cued letter (partial report). The role of VA span in reading speed development was investigated by Bosse and Valdois [21] in a cross-sectional study of 417 elementary school children. In line with MTM model predictions, they found that VA span predicted both reading accuracy and reading speed independently of phonological skills and verbal short-term memory in first, third and fifth grade children. Furthermore, reduced VA span and reduced reading speed co-occur in dyslexic children with no phonological deficit [25]. In addition, case studies [23], [24] have shown that dyslexic children with reduced VA span exhibit word reading length effects, thus reduced reading speed. According to the VA span deficit hypothesis of developmental dyslexia [25], poor visual attention capacity explains the co-occurrence of poor VA span and poor reading performance. VA span, as measured by report tasks, is taken as a measure of general visual attention capacity. In this account, VA span therefore relates to the maximum amount of visual information in a multiple element display that can be encoded *in a limited time*.

Visual attention can be considered as a capacity limited process [27], [28]. According to race models [29], it is limited by two factors: visual *processing* capacity and visual *storage* capacity. Visual *processing* capacity relates to the amount of attentional resources that are available for visual processing. It can be modeled as a finite visual processing speed [30]. Visual *storage* capacity relates to the amount of visual information that can be stored in short-term memory, regardless of available processing time. Storage capacity is usually referred to as visual short-term memory (VSTM) capacity. Empirical evidence converges towards a fixed capacity for VSTM [31]–[33], but see [34].

In order to better understand the relationship between VA span, reading speed and visual attention capacity, one needs to quantify visual *processing* capacity and *storage* capacity. In the Theory of Visual Attention [35], these visual attention capacity limits are modeled by two independent parameters. TVA is a computational model of parallel visual attention according to which visual elements are processed in parallel and compete for a limited number of slots in a short-term memory store (VSTM). Visual processing entails accumulating evidence that a visual object belongs to a specific category (e.g.: ‘blue object’, ‘letter A’). Once enough evidence has been accumulated and a categorization has been made, the visual object is recognized and can enter the store. Since the VSTM store is capacity limited, only those elements that finish processing first are recognized. Once all slots in VSTM are filled, no additional visual object can be consciously recognized (and thus reported). In TVA, visual processing capacity relates to available resources for element sensory processing and is modeled as visual processing speed. Visual storage capacity relates to the number of available slots and is modeled as VSTM capacity. These factors are mathematically defined within TVA as parameters *C* (Visual processing speed) and *K* (VSTM capacity). Individual parameter values are derived by combining behavioral results from whole and/or partial report paradigms and TVA model equations [36]. TVA not only accounts for a wide range of visual selective attention data but has also been used to assess visual attention capacity in both healthy and neurologically impaired adults (see [37] for a review). Furthermore, TVA has previously been successfully used to identify visual attention deficits in dyslexic readers [38], [39].

We will use TVA to specify relationships between individual differences in VA span, visual attention capacity and reading speed in eight and nine year-old children. VA span, as defined by Bosse et al., refers to the number of elements in a visual array that can be *processed* simultaneously. It will be measured as the mean number of letters reported in the VA span task. TVA parameters quantify general visual attention capacity. VSTM capacity *K* refers to the number of elements that can be *stored* in short term memory. Visual processing speed *C* refers to the total amount of processing resources that is divided between all elements in the visual field.

First, we will investigate whether VA span is modulated by visual attention capacity. Behavioral [40] and neural [41]–[43] data show that visual processing of letter strings recruits specialized perceptual processes. Part of this neural tuning for print seems to be specific to *horizontal* letter strings [8]. We will use the term “perceptual specialization” to refer explicitely to these configuration-specific (horizontal string) processes. Since this perceptual specialization develops with reading acquisition [9], [13], [14], it could be the main visual factor modulating VA span scores. This would argue against general visual attention capacity as a predictor of reading performance in learning-to-read children. To rule out perceptual specialization (tuning for horizontal letter strings) as the mechanism responsible for the VA span – reading speed link, we chose to estimate TVA parameters with a circular letter array. Since specialized perceptual processing of letter strings is limited to familiar print-like horizontal layouts [8], [44], it should not be relevant for a circular layout. Accordingly, our estimates of TVA parameters should be modulated by visual attention capacity and not perceptual specialization. If VA span indexes visual attention capacity and not perceptual specialization, then TVA parameters should predict VA span scores.

TVA has previoulsy been used to specify the putative role of visual attention in poor reading ability [38], [39]. Dubois et al. [39] showed that visual processing speed was reduced for two VA span impaired dyslexic children by about 44%. In line with these results, Stenneken et al. [38] reported a 26% average reduction of visual processing speed for a group of high-achieving dyslexic adults. Co-occurring deficits in visual processing speed and reading speed are compatible with predictions of the MTM model, but conclusions on a general relationship between these factors in normal-reading children remain purely speculative. In this study, we will use TVA to investigate whether individual differences in visual attention capacity predict individual differences in reading speed.

**Figure 1:**
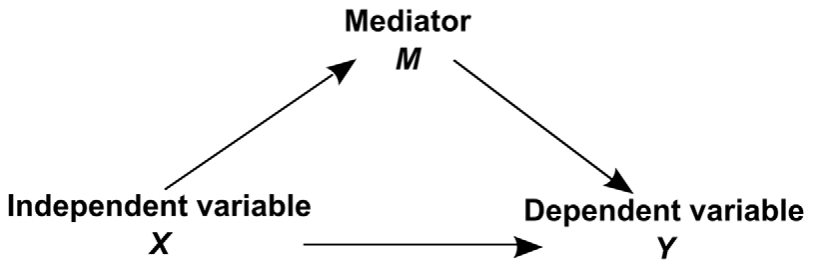
Schematic description of a mediation model. In a mediation model, the effect of an independent variable *X* on a dependent variable *Y* is mediated through the mediator variable *M*.

Bosse and Valdois [21] posit that visual attention capacity explains the linear relationship between VA span and reading performance. Previous studies have ruled out phonological processes (e.g., verbal recoding of letter names or verbal short-term memory) as critical explanatory factors [22], [45], [46]. The relationships between visual attention capacity, VA span and reading speed have however not been investigated. The MTM model posits that visual attention capacity modulates the maximum size of the visuo-attentional window. In turn, the size of the visuo-attentional window would modulate reading speed. We used mediation analysis [47] to test this hypothesis. Mediation analysis specifies the relationship between an independent variable *X* and a dependent variable *Y* by explaining the mechanism by which *X* affects *Y*. In a mediation model, a third variable, the mediator variable *M*, “transmits” the effect of the independent variable *X* on the dependent variable *Y* (see Figure 1). We will test whether VA span is a mediator of the relationship between visual attention capacity and reading speed.

To summarize, in this study we explore how individual differences in reading speed, VA span and visual attention capacity are related in typically developing children of elementary school age. In order to limit the amount of age-related co-variation between variables of interest, we limited the children's age range to 8 and 9 year olds. VA span is estimated using the seminal 5-consonant VA span task [21], [23], [25], [48]. Visual attention capacity parameters are estimated by fitting data from a circular array whole report task to TVA equations [36]. Our predictions pertain to links between TVA parameters and both VA span and reading speed. First, we hypothesize that VA span is constrained by visual attention capacity. We expect visual processing speed *C* and VSTM capacity *K* to predict VA span scores. Second, we hypothesize that reading speed development is modulated by visual attention capacity. Both VA span and TVA parameters should predict reading speed. Finally, we hypothesize that VA span mediates the effect of visual attention capacity on reading speed.

## Methods

### 1 Participants

This research was approved by the local ethics committee and conducted according to the principles expressed in the Declaration of Helsinki. Forty-nine eight and nine year-old children took part in this study (see Table 1). They were recruited from local Grenoble public schools. Children and their legal guardian gave written informed consent before taking part in the experiment. All children were native French speakers and had normal or corrected to normal vision. Screening ensured that children with learning disabilities, cognitive deficits or sensory impairments were excluded.

**Table 1:** Descriptive statistics for all variables.

	Mean	95% CI	Range
Age	108	106–110	97–119
Reading age	107	103–111	86–138
VA span	4.1	3.9–4.2	2.8–5
Visual processing speed *C*	24.6	22.5–26.6	9.3–41.4
VSTM capacity *K*	3.6	3.5–3.8	2.4–4.8
Reading speed	80	74–86	37–130

Age and reading age are reported in months. VA span is reported in number of letters. Units for visual processing speed and VSTM capacity are number of elements per second and number of elements. Reading speed is reported in words per minute.

### 2 Experimental Tasks

#### 2.1 Reading Speed Assessment

Text reading speed (words per minute) was assessed using the “Alouette Reading Test” [49]. This test requires participants to read aloud a 265 word text as quickly and accurately as possible within a 3-min time limit. The text includes unfamiliar words and is structured so as to avoid lexical knowledge-based guessing. Reading speed was computed by dividing the number of words read by time in minutes. This test was also used to assess reading age by comparing a composite score based on both speed and accuracy to normative data from a large sample of French children. Reading age data was used to check that participants' reading ages were contained between the 5^th^ and 95^ th^ percentile values for their chronological age. One participant did not meet this criterion and was excluded from the analyses.

#### 2.2 Visual-Attention span Assessment

VA span was estimated using a 5-consonant global report task (i.e. the “VA span task”), used in a number of previous studies (e.g., [21], [25]). Twenty random five-letter strings were built up from 10 consonants (BPTFLMDSRH), each letter being used 10 times and appearing twice in each position. Stimuli strings contained no repeated letters and never matched the skeleton of a real word. Letters were presented in upper case (Geneva) in black on a white background. Each letter subtended a vertical visual angle of 0.7°, and the distance between adjacent letters was of 0.57°. Each trial began with the display of a fixation point for 1000 ms, followed by a 50 ms blank screen. A letter string was then displayed for 200 ms, followed by a blank screen. Participants were asked to report verbally as many letters as possible. They carried out 10 training trials with feedback and 20 experimental trials with no feedback. The dependent measure was the average number of letters accurately reported (identity, not location) per trial (max = 5).

#### 2.3 TVA whole report task

**Figure 2:**
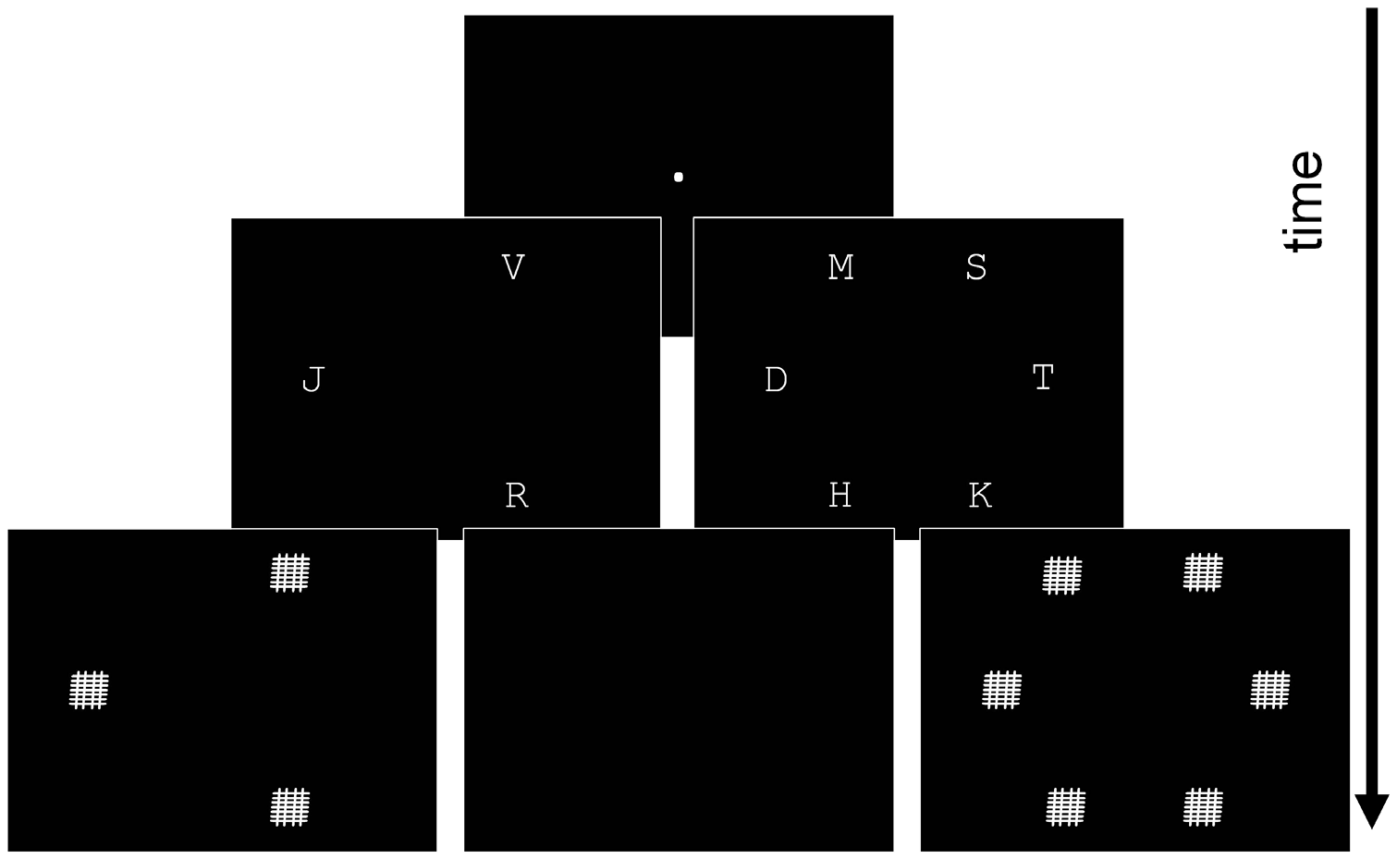
Schematic diagram of the TVA whole report task procedure.

Whole report paradigms that display several visual elements at varying exposure durations are required for TVA-based modeling. Both umasked and post-masked trials may be included. A 3 and 6-letter whole report task (i.e. “the TVA task”, see Figure 2) was run under the MATLAB environment (MathWorks Ltd) using software from the Psychtoolbox [50]–[52]. Stimuli were presented on a Dell CRT monitor with a 100 Hz refresh rate. Participants were asked to fixate a central white dot on a black screen. As soon as fixation was deemed appropriate, the experimenter launched the trial. Either 3 or 6 uppercase white letters, randomly drawn without replacement from a set of 15 consonants (BCDFHJKLMNPRSTV), were briefly (20–200 ms) displayed. Letter height was 0.7° of visual angle. Each letter was presented at one of six equally distant positions drawn at the circumference of virtual circle (0°, 60°, 120°, 180°, 240°, 300°; radius of the circle: 3.3°), ensuring sufficient distance between neighboring letters to minimize crowding effects. Positions for the 3-letter condition were pseudo-randomized. Stimulus presentation was immediately followed either by a blank screen or a 500 ms pattern mask displayed on each position that had previously held a letter. After presentation, participants were asked to verbally report all letters they had seen. Report was unspeeded and participants were asked to refrain from guessing.

Three different exposure durations were used. To avoid useful eye movements, the longest exposure duration was set at 200 ms for all participants. The other two durations were estimated individually before the experiment. For optimal modeling of parameter *C*, it is necessary for the minimum display duration (*D_min_*) to be close to perceptual threshold *t_0_*. Therefore, *D_min_* was estimated individually before the experiment and set to the 50% accuracy threshold for single peripheral letter identification (mean: 46 ms, range: 20–110). This threshold was measured using the improved QUEST algorithm [53] on 20 trials. An intermediate display duration was defined by adding 70 ms to *D_min_*. All three exposures were used in masked conditions while the minimum *D_min_* and maximum (200 ms) were also used in an unmasked condition. Unmasked conditions allow a prolonged *effective* exposure duration due to visual persistence [33]. This prolonged maximum exposure ensured that participants reached near ceiling report performance. The two display sizes (3 and 6 letters) and 5 effective exposure durations per display size (*D_min_* masked and unmasked, *D_min_* +70 ms masked, and 200 ms masked and unmasked) added up to a total of 10 conditions. Blocks of 50 trials (5 trials per condition, randomly ordered) were defined. In order to robustly estimate TVA parameters, children carried out 15 trials per condition [54] (i.e., 3 blocks interleaved amongst the other experimental tasks).

### 3 TVA parameter estimation

We used the TVA framework to assess VSTM capacity and visual processing speed. In order to derive quantitative values for these parameters from behavioral results, data from the TVA task were fitted to model equations.

For each participant, a maximum-likelihood algorithm was used to fit the raw data to an exponential growth function (see [35] for a complete presentation of the mathematical framework; and [36] for the fitting procedure and software). Four parameters were estimated. The first two are our parameters of interest: (i) *C* (number of elements processed per second), the visual processing speed; and (ii) *K* (number of elements), VSTM storage capacity. The last two are *t_0_* (ms), the perceptual threshold beneath which no letter is encoded; and *µ* (ms), the iconic memory buffer estimated from the accuracy difference between masked and unmasked trials. Parameter reliability was assessed by bootstrap analyses (500 bootstrapped samples).

**Figure 3:**
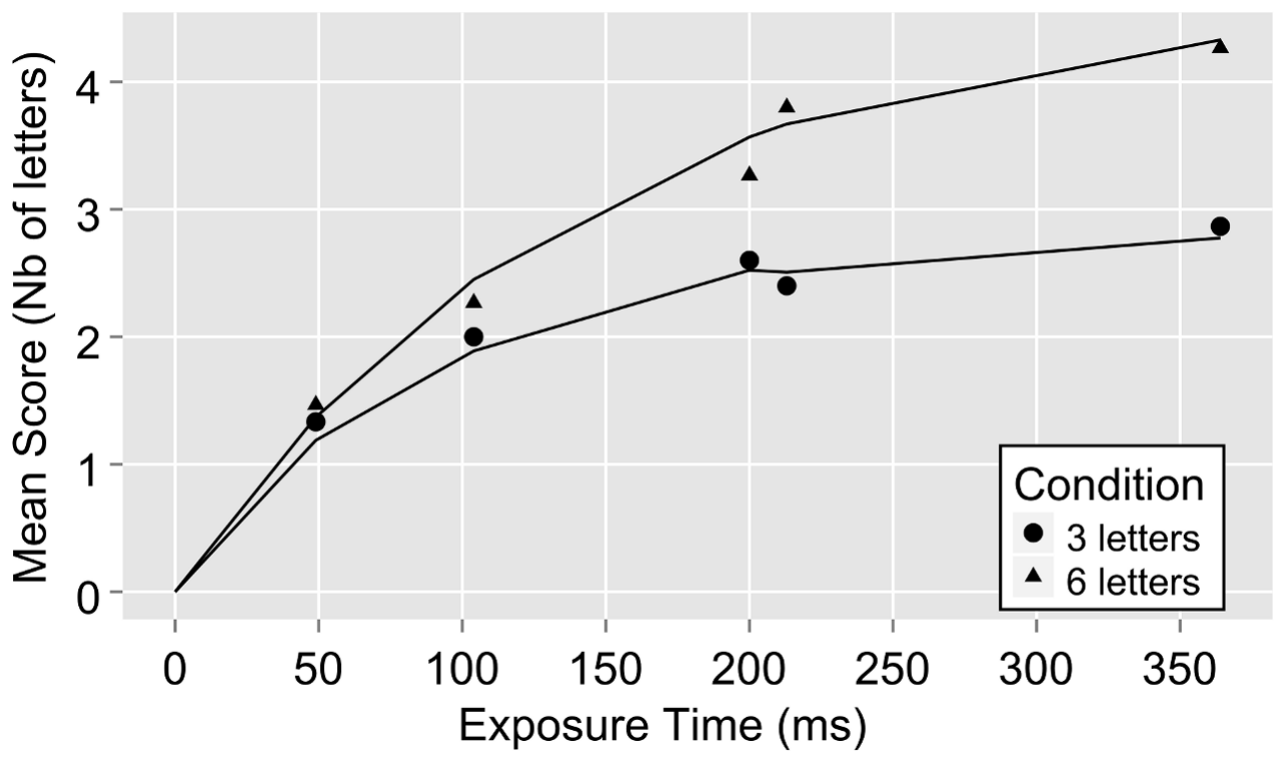
Performance of a typical participant for the TVA whole report task. The mean score (mean number of correctly identified letters) is plotted against exposure time. The solid lines are the fitted curves after TVA analysis of the behavioral data for each condition.

Raw performance and the theoretically derived function are plotted for a representative participant in Figure 3. *C* is captured by the slope of the exponential growth function at its origin (*t_0_*, 0). VSTM capacity *K* is captured by the function's asymptote, when the number of presented objects is large enough to fill all VSTM slots (as in our 6 letters condition).

### 4 Mediation analysis

**Figure 4:**
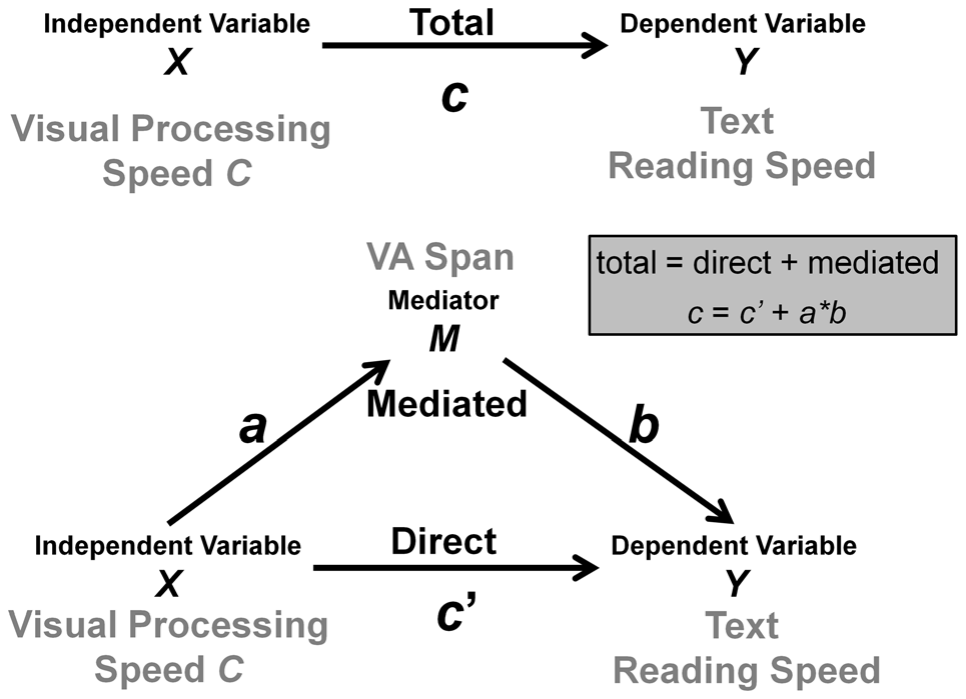
Schematic descriptions of total and mediated effects of visual processing speed on reading speed. (Top) Schematic description of the total effect of visual processing speed on reading speed, with path *c* estimated from regression Equation (1). (Bottom) Schematic description of the mediated effect of visual processing speed on reading speed through VA span. Mediated effect paths *a* and *b* are respectively estimated by regression Equations (2) and (3). Path *c*' is estimated by Equation (3).

We used mediation analysis [47] to test whether VA span mediated the effect of visual attention on reading speed. Mediation analysis specifies the relationship between an independent variable *X* and a dependent variable *Y* by explaining the mechanism by which *X* affects *Y*. In a mediation model, a third variable, the mediator variable *M*, “transmits” the effect of the independent variable *X* on the dependent variable *Y* (see Figure 4). *X* influences *M* which, in turn, influences *Y*. The effect of *X* on *Y* (path *c* in Figure 4) is referred to as the total effect. The effect of *X* on *Y* through *M* (paths *a* and *b* in Figure 4) is referred to as the mediated effect. The effect of *X* on *Y* in the mediated model (paths *c*' in Figure 4) is referred to as the direct effect.

In this study, we proposed that visual attention capacity (as measured by TVA parameters) exerts an influence on “reading speed” (as measured by text reading speed) through the mediating variable “VA span” (as measured by the VA span task). We hypothesize that individual differences in visual attention capacity predict individual differences in VA span. These individual differences in VA span would then predict subsequent individual differences in reading speed.

We chose to use two of the several possible methods to assess the size and significance of a mediated effect [47], [55]. First, we ran an analysis using the classic causal-steps approach [56]. This method, although widely used, has several drawbacks [55]. It may fail to detect a mediation effect if effect sizes of paths *a* and *b* are small. It also does not quantify the strength of the mediated effect. For these reasons, we implemented a bootstrap approach to estimate the mediated effect (i.e., *a*×*b*) [57], [58], as recommended by current literature [59].

#### 4.1 Causal-steps analysis

To specify how visual attention capacity, VA span and reading speed are related, a three-step multiple regression approach is necessary [56] as illustrated in Figure 4. First, variation in the visual processing factor (in this example, visual processing speed *C*) should significantly predict variation in reading speed (path *c* in Figure 4). Second, variation in visual processing speed should also significantly predict variation in VA span (path *a* in Figure 4). Third, VA span should significantly predict variation in reading speed when visual processing speed is also included in the regression model (path *b* in Figure 4). These predictions are tested using three regression models:
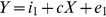
(1)

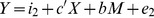
(2)

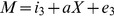
(3)


In the second regression model, if coefficient *c*' is not significant, mediation is said to be total [56].

#### 4.2 Estimation of the mediated effect

Mediation can also be assessed by testing the significance of the indirect or mediated effect computed as the product of regression coefficient estimates 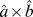 [47]. The most accurate way of testing this significance is by building confidence intervals for the mediated effect using a bootstrapping approach. The available data is resampled with replacement 1,000 times and regressions (2) and (3) are run for each resampling. The sampling distribution of *a x b* is built and 95% confidence intervals (95% CI) are constructed from the resampled data without making any parametric assumptions. The mediation effect is deemed to be significant if the 95% CI does not contain 0. As a final step in the analysis, we estimated the effect size of our mediated effect using *κ^2^* [60]. Preacher & Kelley [60] define *κ^2^* as the proportion of the maximum mediated effect that could have occurred given the data and the design (i.e., the observed mediated effect divided by the maximum possible mediated effect). A point estimate of *κ^2^* as well as 95% CI were computed by bootstrap.

## Results

### 1 TVA fits and data screening

Individual TVA model fit was deemed appropriate if the Pearson product-moment correlation coefficient between the empirical and theoretical (fitted) mean scores for each condition was above.75. All participants' correlation coefficicents were above this cutoff. The average correlation coefficient was .88 (range: .79–.93, computed after applying a Fisher *z*-transform to the correlation coefficients). Individual parameter reliability was deemed appropriate (bootstrapped distributions for each parameter were close-to-normal with small standard deviations). The average *t_0_* value was 4 ms (range: 0–25), and the average *µ* value was 184 ms (range: 93–382).

Data were screened for outliers before analysis. We computed participants' *z*-scores for each experimental measure (reading speed, VA span, visual processing speed *C* and VSTM capacity *K*). One participant whose absolute *z*-score on one variable was above 2.5 was excluded from further analysis. The final sample included 47 participants (96% of the initial number of participants). Descriptive statistics for all experimental measures are presented in Table 1.

### 2 Relationship between age, reading speed, VA span, and TVA parameters

Four variables of interest were measured: VA span, visual processing speed *C*, visual short term memory capacity *K* and reading speed. A broad overview of the relationship between the different variables of interest is given by pair-wise Pearson correlation coefficients, for both full correlations between variables and partial correlations controling for the effects of age (see Table 2). Age was significantly correlated to all variables except VSTM capacity *K*. VA span was significantly correlated to both TVA parameters as well as to reading speed. There was a significant correlation between parameters *C* and *K*. Finally, there was a medium correlation between *C* and reading speed and a weak correlation between *K* and reading speed. All partial correlations were significant.

**Table 2:** Correlation matrixfor age, VA span, TVA parameters and reading speed.

	VA span	*C*	*K*	Reading speed
Age	.30	.39**	.19	.40**
VA span		.67***	.67***	.56***
*C*	.63***		.54***	.50***
*K*	.66***	.51***		.34*
Reading speed	.51***	.41**	.29*	

Above diagonal: correlation coefficients (Pearson's *r*) for age, VA span, TVA parameters and reading speed.

Below diagonal: partial correlation coefficients for VA span, TVA parameters and reading speed controlling for age.

Significance levels: *: *p*<.05, **: *p*<.01, ***: *p*<.001.

We used multiple regression analyses to test our different hypotheses. In order to factor out confounding effects of collinearity between age and our different other variables, age was included as a regressor in all the following models. To investigate whether TVA parameters predict VA span, we computed a multiple linear regression model with VA span as the dependent variable and TVA parameters *C* and *K* as regressors (Model 1). To investigate whether VA span predicts reading speed, we computed a simple linear regression model with reading speed as the dependent variable and VA span as the sole regressor (Model 2). To investigate whether visual attention capacity predicts reading speed, we computed a multiple linear regression model with VA span as the dependent variable and TVA parameters *C* and *K* as regressors (Model 3).

**Figure 5:**
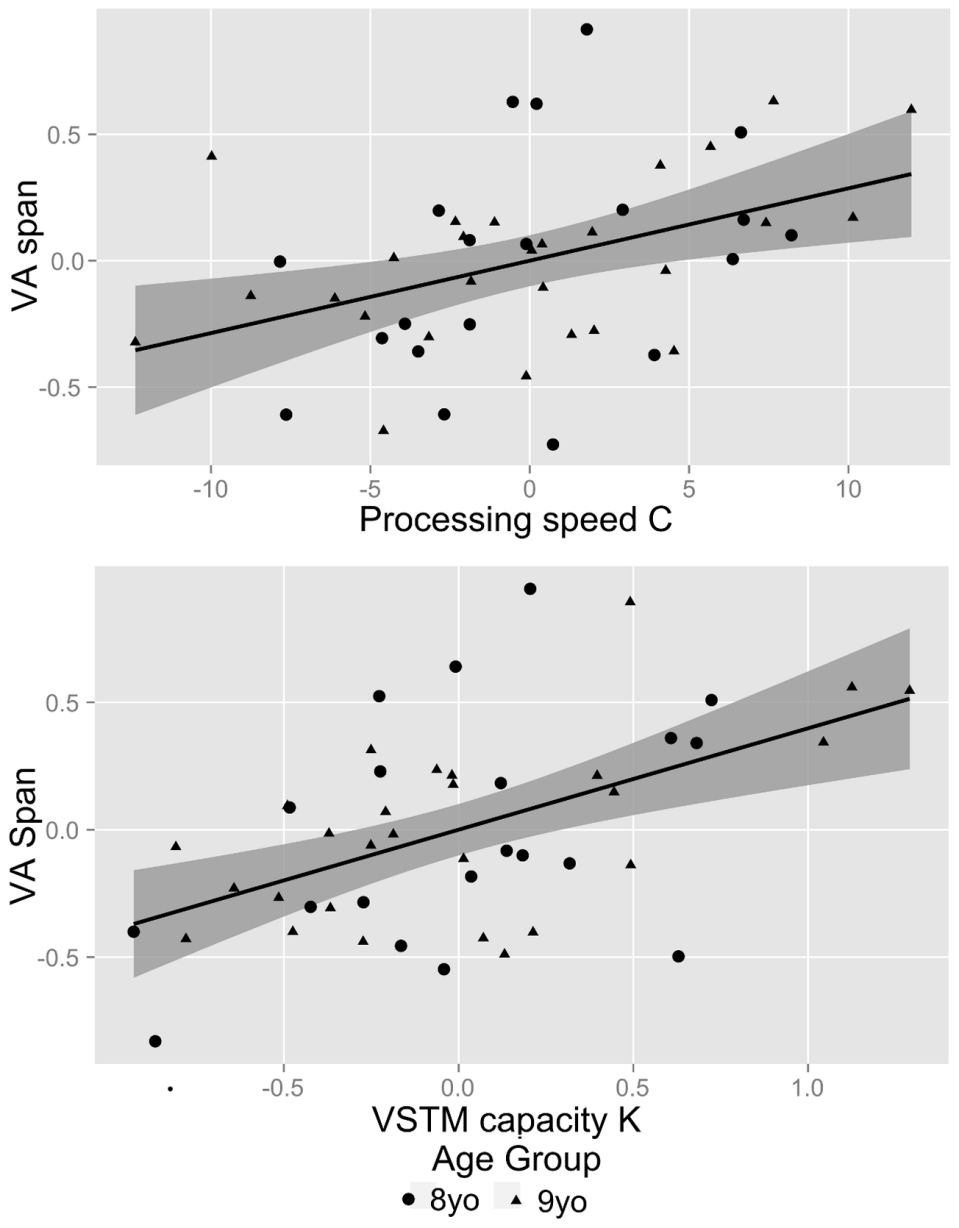
Partial regression plots and sample regression model for regression *VA span*  =  *age* + *C* + *K*. (Top) Partial regression plot for the effect of visual processing speed *C* on VA span, controlling for age. The residuals of the regression of VA span on VSTM capacity *K* and *age* are plotted against the residuals of the regression of visual processing speed C on VSTM capacity *K* and *age.* (Bottom) Partial regression plot for the effect of VSTM capacity *K* on VA span. The residuals of the regression of VA span on visual processing speed *C* and *age* are plotted against the residuals of the regression of VSTM capacity *K* on visual processing speed *C* and *age.* The solid lines represent the regression line and the shaded areas represent the regression lines' 95% confidence intervals.

For regression model 1 (Figure 5), This full model explains 59% of the variance in VA span (*F*(3,43)  = 20.3, *p*<.0001). Both *C* (*p*<.01) and *K* (*p*<.001) contribute significant variance to VA span, but age does not (*p* = .63). The slope of the partial regression line for visual processing speed indicates that if age and *K* are held constant, an increase of ten elements per second results in an increase in 0.3 letter in VA span. Similarly, if *C* and age are held constant, an increase of one element in VSTM capacity results in an increase in 0.4 letters in VA span.

**Figure 6:**
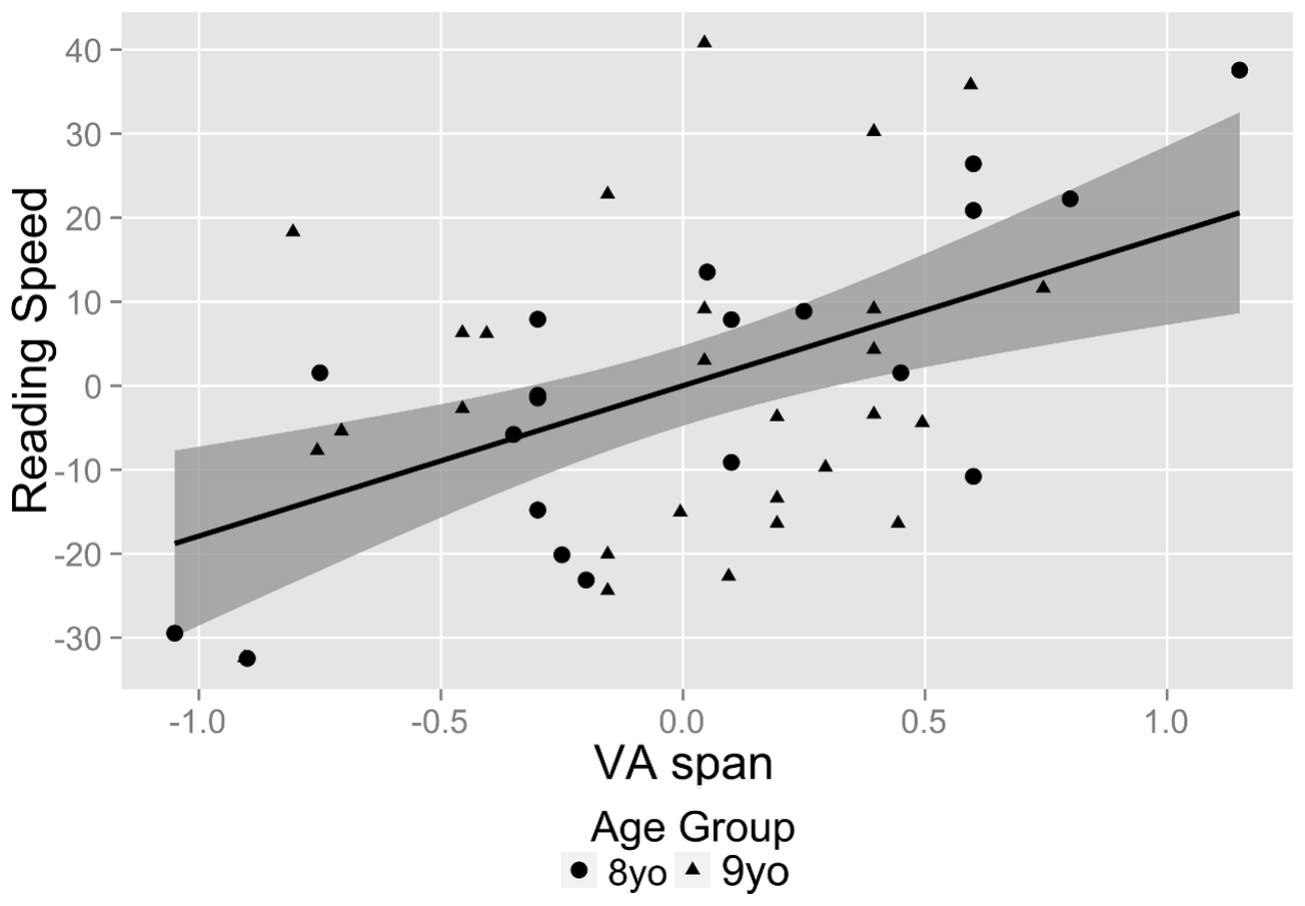
Regression plot and sample regression model for regression *Reading Speed*  =  *VA span*. The residuals of the regression of *reading speed* on *age* are plotted against the residuals of the regression of *VA span* on *age*. The solid line represents the regression line and the shaded area represents the regression line's 95% confidence interval.

Regression model 2 (Figure 6) explains 38% of the variance in reading speed (*F*(2,44)  = 13.3, *p*<.0001). Both age (*p*<.05) and VA span (*p*<.001) contribute significant variance to reading speed. The slope of the partial regression line for VA span indicates that if age is held constant, an increase of one unit in VA span results in an increase of 19.0 wpm in reading speed. If VA Span is held constant, a one month increase in age results in a reading speed increase of 0.8 wpm.

**Figure 7:**
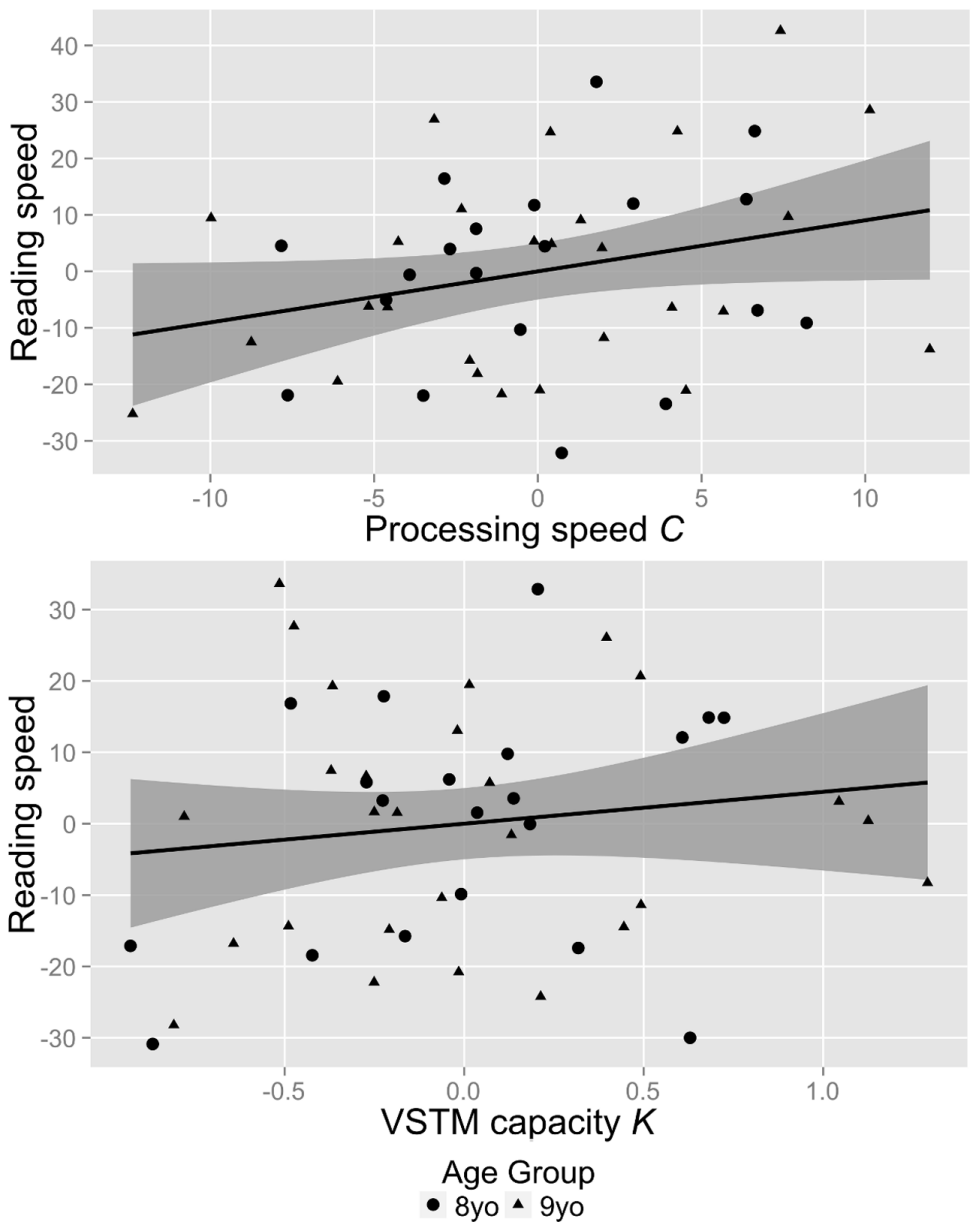
Partial regression plots and sample regression model for regression *Reading Speed*  =  *age* + *C* + *K*. (Top) Partial regression plot for the effect of visual processing speed *C* on reading speed controlling for age. The residuals of the regression of *reading speed* on VSTM capacity *K* and *age* are plotted against the residuals of the regression of visual processing speed *C* on VSTM capacity *K* and *age.* (Bottom) Partial regression plot for the effect of VSTM capacity *K* on reading speed. The residuals of the regression of *reading speed* on visual processing speed *C* and *age* are plotted against the residuals of the regression of VSTM capacity *K* on visual processing speed *C* and *age.* The solid lines represent the regression lines and the shaded area represent the regression lines' 95% confidence intervals.

For regression model 3 (Figure 7), 31% of the variance in reading speed is explained by TVA parameters and age (*F*(3,43)  = 6.4, *p*<.01). The only variable that contributes significant variance to reading speed is visual processing speed C (*p*<.05; age: *p* = .09; VSTM capacity *K*: *p* = .53). The slope of the partial regression line for *C* indicates that if age and *K* are held constant, an increase of one unit in visual processing speed *C* results in a reading speed increase of 1.1 wpm.

### 3 Mediation Analysis

Results from our regression analysis show that only one component of visual attention capacity, visual processing speed *C*, modulates reading speed. Therefore, we considered a single mediation model with visual processing speed *C* as the independent variable. The dependent variable was reading speed. The mediator variable was VA span. The classic causal-step approach [56] and the now recommended bootstrapping approach [61] were carried out. To ensure that results from the mediation analysis were not influenced by a common effect of age on all variables of interest, age was added as a regressor to all mediation regression models. The regression models used in both the causal steps and bootstrap methods were thus the following:
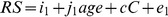
(1')

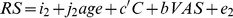
(2')

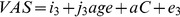
(3')


**Figure 8:**
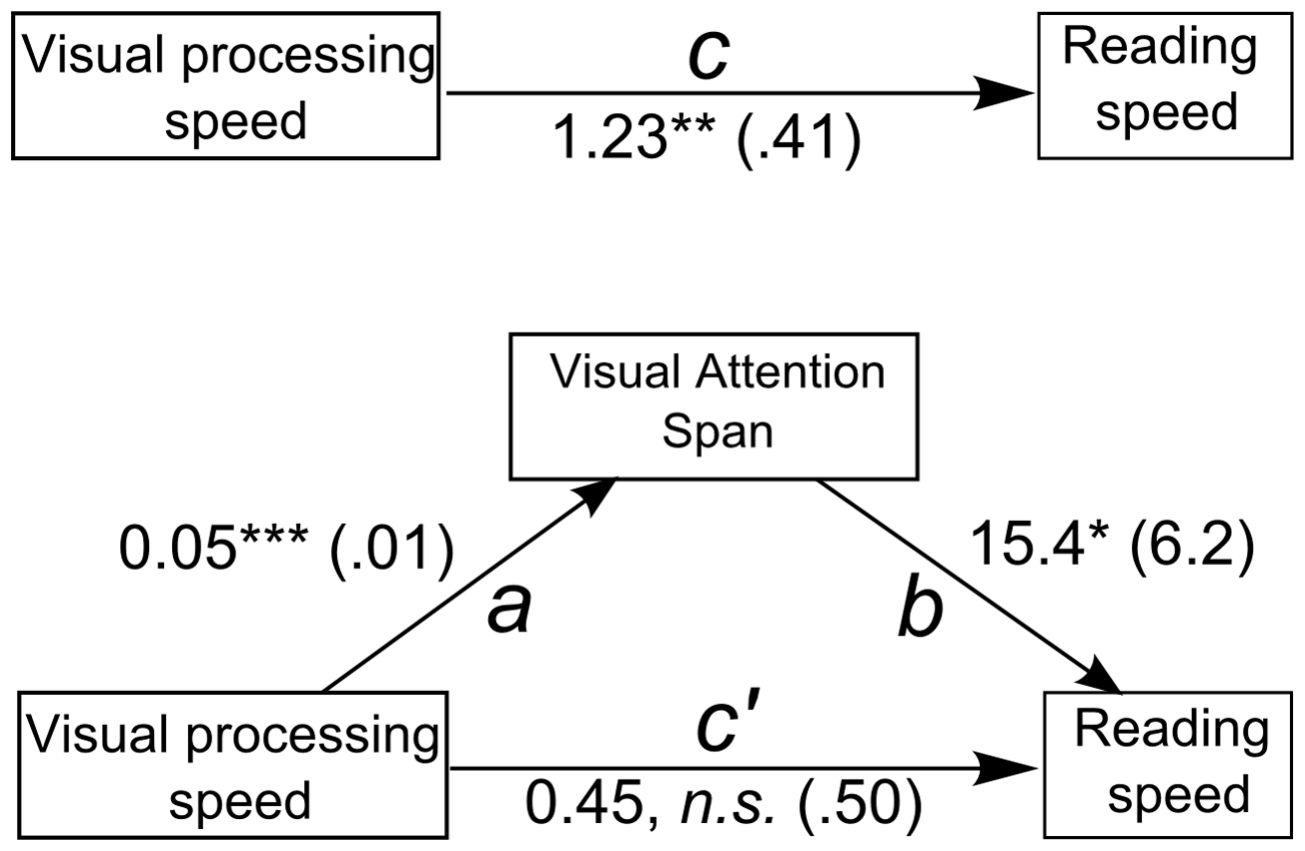
Schematic diagram of mediation analysis results. Differences in VA span significantly mediated differences in visual processing speed. Path values are unstandardized regression coefficients with standard errors in parentheses. Significance levels are as follows: *: *p*<.05, **: *p*<.01, ***: *p*<.001, *n.s.* non-significant.

According to the causal-step approach (see Figure 8 for regression coefficients and statistical significance), individual differences in VA span significantly mediated the effect of visual processing speed on reading speed. Visual processing speed *C* significantly predicted reading speed (path *c* was significant). Visual processing speed *C* also significantly predicted VA span (path *a* was significant). VA span still significantly predicted reading speed once the effects of *C* have been taken into account (path *b* was significant). However, the effect of visual processing speed *C* became non-significant after accounting for differences in VA span (path *c*' non-significant). The amount of variance in reading speed explained by visual processing speed *C* was therefore significantly reduced when differences due to VA span were taken into account (compare paths *c* and *c*' in Figure 8). A non-significant direct effect path (*c*') suggests that VA span completely mediates the effect of visual processing speed on reading speed [56].

A point estimate and confidence intervals representing the mediated effect were derived from the bootstrapped product of paths *a* and *b*. The 95% BCa Confidence Interval for the indirect effect *a* x *b* did not contain 0 (point estimate  = 0.77, BCa  =  [0.13–1.67]). This result is consistent with results from the causal-steps approach: VA span significantly mediated visual processing speed related differences in reading speed. In order to estimate the size of our mediated effect, we derived a point estimate and 95% BCa CIs for *κ^2^* from 1000 bootstraps using the MBESS R package [62]. Within Cohen's guidelines [63], the derived effect size was in the medium range (*κ^2^*  = .22, BCa  =  [.05–.44]).

## Discussion

Our study explored interactions between VA span (number of visual elements that can be extracted from a visual array in parallel), visual attention capacity as defined by TVA (visual processing speed *C* and VSTM capacity *K*) and reading speed in a group of normally developing eight and nine year old children. Our first hypothesis was that if VA span is modulated by visual attention capacity, then visual attention parameters *C* and *K* should predict VA span. Our second hypothesis was that if reading speed is modulated by visual attention capacity, then visual attention parameters *C* and *K* should predict reading speed. Our last hypothesis was that VA span mediates the effect of visual attention capacity on reading speed.

### 1 Does visual attention capacity predict VA span?

Both visual processing speed and VSTM capacity predict VA span, even when the effects of age are partialled out. Visual processing speed and VSTM capacity could be important limiting factors in VA span performance. According to TVA, visual processing speed indexes available visual processing capacity for multiple element displays. Participants with high *C* values have more processing ressources available to allocate to each element in the visual field than participants with low *C* values. Therefore, elements will be processed faster for high multiple element *C* values than for low *C* values. As a consequence, low *C* values reduce the number of visual elements that finish processing within a limited 200 ms time window. Participants with low visual processing speeds recognize fewer letters within the 200 ms presentation window than participants with higher processing speeds. Consequently, they report fewer letters for each trial and their total score is lower. Similarly, children with a smaller VSTM capacity have fewer available memory slots to store encoded letters than those with a larger VSTM capacity. Consequently, they report fewer letters and their total score is lower.

VA span is measured using a horizontal letter string as stimuli; therefore it could be argued that it mainly reflects perceptual expertise for horizontal letter strings. However, specialized processing for horizontal letter strings is disrupted when letters are displayed in unfamiliar spatial configurations [8]. Thus, if VA span performance solely reflects perceptual specialization, we expect it to vary independently from TVA parameters estimated with a circular letter array. In contrast, TVA parameters *C* and *K* predict 59% of VA span variability. This supports the hypothesis that VA span is modulated at least in part by visual attention capacity limits unrelated to perceptual specialization for horizontal letter strings.

### 2 Does visual attention capacity predict reading speed?

According to MTM model predictions, VA span should predict reading speed independently from age. Indeed, results show that VA span explains a significant part of reading speed variance after the effect of age has been partialled out, in agreement with the literature [21]. A large VA span ensures that more letters are processed and identified simultaneously, favoring the fast, global reading procedure. Conversely, a small VA span limits the amount of visual information available for word identification, favoring a slower analytic procedure based on sublexical unit processing.

We hypothesized that reading speed is modulated by visual attention capacity. Indeed, visual processing speed *C* predicts reading speed, even when the effect of age is partialled out. Children with high visual processing speeds are faster readers than children with low processing speeds. In contrast, VSTM capacity does not significantly predict reading speed: children with large VSTM capacity do not tend to be faster readers than children with small VSTM capacity. Modulation of reading speed by visual processing speed and not VSTM capacity is consistent with results from Dubois et al. [39] and Stenneken et al. [38]. Comparing dyslexic and normal readers, they showed that while dyslexia was consistently associated with reduced visual processing speed, VSTM capacity was generally preserved. Here, we go further and show that individual differences in visual processing speed predict individual differences in reading speed. This result could relate to visual constraints on reading performance. Children with low multiple element visual processing speeds need more time to extract the necessary visual information for parallel multiple letter identification. As a consequence, their visual word recognition is slowed down.

### 3 VA span mediates the effect of visual processing speed on reading speed

Visual processing speed *C* predicts both VA span and reading speed. These results are compatible with the hypothesis of visual processing speed as an explanatory factor behind the relationship between VA span and reading speed. Using mediation analysis, we investigated whether visual processing speed influences reading speed through its effect on VA span. To rule out any spurious common influence of age on our variables of interest, we added it as a covariate to all models of the mediation analysis. Both the causal-steps and bootstrap analyses confirm that VA span mediates the effect of visual processing speed on reading speed. The causal-steps analysis is consistent with total mediation by VA span (path *c*'  = 0). Furthermore, the magnitude of the observed indirect effect (*κ^2^)* was 18% of the maximum possible indirect effect given our data and model.

This result gives us additional insight on the mechanism through which visual processing speed could modulate reading speed. Visual processing speed *C* represents the total amount of resources available to process multiple element visual arrays. In the VA span task, visual processing speed determines the total amount of resources available to process the letter string and thus the resources available for sensory processing of each letter. Indeed, all letters are processed in parallel, but each letter must share available resources with the other letters. Individuals with higher multiple element visual processing speeds thus process letters faster than individuals with lower multiple element visual processing speeds. As a consequence, more letters finish processing within the allotted time frame. This leads to a larger number of correctly reported letters, hence a larger VA span estimate. In turn, a large VA span means that when fixating a word, a sufficient number of letters is identified in a single glance. According to the MTM model, this favors a fast global procedure of word identification. Within this framework, VA span is the critical mechanism to understand the role of visual attention capacity in reading.

An alternative explanatory mechanism for the relationship between VA span and reading speed could be perceptual specialization for horizontal letter strings. Perceptual specialization modulates reading performance in children [14]. If it also modulates performance on the VA span letter report task, it could explain the linear relationship between reading speed and VA span. While our mediation analysis cannot completely rule out the possibility that neural tuning for individual letters accounts for part of this relationship, it suggests that VA span and visual processing speed account for the same variance in reading speed. Since visual processing speed is independent from perceptual specialization, this result supports the hypothesis that this perceptual specialization is not the sole factor explaining the relationship between VA span and reading.

The role of VA span as a mediator between visual processing speed and reading speed is critical in specifying the underlying causes of VA span deficits in developmental dyslexia [25]. Dubois et al. [39] previously reported co-occurring deficits of reading speed, VA span and multiple element visual processing speed in two dyslexic children. In light of our results, it seems plausible that just as VA span size mediates the effect of visual processing speed on reading speed, reduced VA span size in dyslexia could mediate the effect of reduced visual processing speed on reading. Reduced visual processing speed would be the core deficit responsible for both reduced VA span and impaired reading. A letter-specific visual processing speed deficit could however also explain these results. However, recent data suggests that the VA span deficit extends to visual processing of character strings made up of digits [46] and unfamiliar characters [22]. Poor performance for non-letter string processing cannot be explained by a letter-specific visual processing speed deficit. Although further research needs to specifically evaluate non-letter visual processing speed in VA span impaired dyslexic children, the available data argues for the existence of a general multiple element visual processing speed deficit.

The development of reading efficiency is driven by different cognitive and linguistic factors [64]–[66]. Regarding the visual front-end of visual word recognition, extensive evidence relates the acquisition of reading skills to increased visual perceptual tuning for print and supports a causal interpretation of this relationship [13]–[15]. We suggest that visual attention could also play an important role in reading speed development, in line with our experimental results. In learning-to-read children, perceptual tuning for print is not fully developed [9], [13], [67]. Letter strings could thus be processed like any multiple element display. Indeed, results on the VA span task carried out with digits are highly correlated to both VA span (measured with letters) and reading performance in normal-reading children [46]. Furthermore, VA span predicts multiple character categorization performance for unfamiliar, non-verbal characters in elementary school children [22]. Finally, a similar posterior parietal brain network seems to be involved in multiple character processing for alphanumeric and non-alphanumeric character strings [68]. Common visual processes could thus drive multiple element and multiple letter (letter string) processing during reading acquisition. In that case, visual processing capacity (i.e., visual processing speed) could modulate multiple letter recognition efficiency and in turn constrain word identification rate.

The main limitations of this study stem from the use of letters as stimuli. First, we have chosen to use multiple letter processing capacity as a proxy for general multiple element processing capacity. Nonetheless, general and letter-specific visual processing capacity could have different growth curves during childhood. There is evidence that general visual processing speed improves during childhood [69], but its putative relationship to letter-specific processing speed has yet to be investigated. In contrast, recent data shows that VA span predicts non-letter multiple character processing [22]. This relationship cannot be explained by a modulation of VA span by letter-specific visual processing capacity. It is, however, compatible with a modulation of both VA span and non-letter multiple character processing by general visual processing capacity. Further research is now needed to assess visual processing speed using non-letter stimuli in order to completely disambiguate between what is general and what is letter specific. Second, our study does not completely identify the direction of the relationship between visual processing speed and reading speed development. The development of reading efficiency might be the driving factor behind the development of multiple letter processing speed. Reading acquisition would then lead to a general improvement of visual processing efficiency [16], [70], which would in turn result in increased multiple element processsing efficiency. If this were the case, we would however expect impaired reading to be systematically associated with poor multiple letter processing. On the contrary, data shows that all dyslexic children do not have a VA span deficit [23], [25], suggesting that reading performance is not the sole predictor of multiple element processing performance. Finally, the limited age range of our group (22 months) does not allow generalization of our results to other age groups. Further research is needed in order to extend our results to the early stages of reading acquisition (6–8 year olds) as well as to the later stages of fluency development (10 years old and up).

This study brings new insights on the influence of visual attention capacity on children's reading speed. First, we showed that VA span is modulated by visual attention capacity. More importantly, we showed that visual processing speed predicts reading speed, and that this effect is mediated by VA span. Our results argue for a role of general visual attention capacity in the visual front-end of reading independently from reading-specific perceptual specialization for horizontal letter strings.
